# Synergistic Effect of Huangqin Decoction Combined Treatment With Radix *Actinidiae chinensis* on DSS and AOM-Induced Colorectal Cancer

**DOI:** 10.3389/fphar.2022.933070

**Published:** 2022-07-06

**Authors:** Jianbo Huang, Tao Jiang, Jingyi Kang, Jiayi Xu, Yiting Dengzhang, Zhengqi Zhao, Chuqi Yang, Mengting Wu, Xiaoping Xu, Guangji Zhang, Zhaohuan Lou

**Affiliations:** ^1^ School of Basic Medical Sciences, Zhejiang Chinese Medical University, Hangzhou, China; ^2^ The Third Affiliated Hospital of Zhejiang Chinese Medical University, Hangzhou, China; ^3^ Key Laboratory of Blood-stasis-toxin Syndrome of Zhejiang Province, Hangzhou, China; ^4^ Laboratory Animal Research Center, Zhejiang Chinese Medical University, Hangzhou, China; ^5^ School of Pharmacy, Zhejiang Chinese Medical University, Hangzhou, China

**Keywords:** colorectal cancer, Huangqin Tang, IL-6/STAT3 signal pathway, T lymphocyte, claudin-1, intestinal flora

## Abstract

**Objective:** To demonstrate the effectiveness of Huangqin decoction (Huangqin Tang in Chinese, HQT) combined with Radix *Actinidiae chinensis* (Tengligen in Chinese, TLG) under the guidance of “dampness–heat theory” in preventing and treating colorectal cancer (CRC) with dampness–heat accumulation and to preliminarily reveal its mechanism.

**Methods**: The mice model of CRC was established by intraperitoneal injection of AOM combined with consumption of 2.5% DSS solution, and celecoxib, HQT, TLG, and their combination (HQT + TLG) were administered at the same time. The physical signs and death of the mice were observed daily. At the end of the experiment, the colorectal tissue was dissected, and the tumor was observed and counted. HE staining and Masson’s staining were used to observe the histopathological changes of colon. Expression levels of TNF-α, IL-6, and IL-10 in colorectal tissue were detected by ELISA, and the expression of TNF-α was observed by immunofluorescence. TUNEL assay was used to observe the apoptosis of tumor tissues, and immunohistochemistry was used to observe the expression of Ki-67 and occludin. The mRNA expression levels of claudin-1, occludin, ZO-1, and IL-6 and IL-17 were detected by RT-PCR, and occludin, ZO-1, NF-κB, and STAT3 protein levels were detected by Western blot. The composition of intestinal flora was analyzed by 16S rRNA.

**Results:** HQT + TLG could significantly reduce the mortality of model mice and improve the intestinal mucosal inflammatory cell infiltration and high-grade intraepithelial neoplasia in model mice. All administration groups show a great reduction in the levels of IL-6 and TNF-α in the colorectal tissues of model mice, and increase in the level of IL-10, the total number of CD3^+^ T cells, the proportion of CD3^+^CD4^+^ T cells, and the ratio of CD4/CD8. HQT and HQT + TLG could significantly change the composition of intestinal flora and increase the abundance of Firmicutes and Patescibacteria.

**Conclusion:** HQT and TLG combination has a good effect on inhibiting AOM-DSS-induced CRC. This function may be related to improving the composition of the intestinal flora, regulating the proportion of T-cell subsets in colorectal lymphoid tissue to improve inflammatory response, and downregulating the expression of claudin-1, inhibiting the activation of IL-6/STAT3 signaling pathway to improving abnormal hyperplasia.

## 1 Introduction

Colorectal cancer (CRC) is one of the most common digestive malignant tumor globally ranked third among cancer-related deaths (Siegel et al., 2017; Cao and Chen, 2019). The incidence and mortality rates of CRC are high in China, especially in Guangdong province (China Anti-Cancer Association, 2022). Currently, inflammatory bowel disease (IBD) such as ulcerative colitis (UC) and Crohn’s disease (CD) is considered to be closely related to the occurrence and development of CRC (Zhang, 2020).

Huangqin decoction (Huangqin Tang in Chinese, HQT), a classic Chinese medicine formula, originates from the “Treatise on Cold Pathogenic Diseases” of Zhang Zhongjing (Zhang, 1955), is composed of *Scutellaria baicalensis* Georgi, *Cynanchum otophyllum* Schneid*.*, *Glycyrrhiza uralensis* Fisch*.*, and *Ziziphus jujuba* Mill. Modern medical research shows that Huangqin Tang can improve intestinal function by inhibiting inflammation (Wang, 2020a) and it can treat ulcerative colitis of damp–heat accumulation type (Wu, 2016) and CRC (Wang, 2015). At the same time, Huangqin Tang can also prevent the UC-associated bowel cancer induced by DSS through the antioxidant pathway (Chen et al., 2016), and its mechanism of action is related to inhibiting the excessive hyperplasia of intestinal epithelium and promoting the apoptosis of tumor cells (Hong-gang, 2018). By some clinical studies, Huangqin Tang can effectively inhibit inflammatory cytokines in serum and promote the recovery of secretion and reabsorption of intestinal mucosa (Yang, 2015). Huangqin Tang combined with chemotherapy has a significant clinical effect in the treatment of colon cancer (Song et Al., 2020) and can alleviate the toxic side effect of chemotherapy drugs on the gastrointestinal tract (Yuan, 2014; Yao, 2017), then enhance the anti-tumor activity of chemotherapy drugs (Zheng, 2011).

Radix *Actinidiae chinensis* (Tengligen in Chinese, TLG), which is the root of *Actinidia arguta* (Sieb. et Zucc) Planch. ex Miq., possesses many functions such as invigorating the spleen and dispelling dampness, clearing away the heat-fire and detoxification, dissipating phlegm, and resolving masses. TLG was found to have antagonistic effects against cancers of the stomach, esophagus, colon, and lung (Wang et al., 2010; Chen et al., 2011; Guo-nong, 2015; He, 2015). Clinically, as a commonly used herb in the clinical treatment of colorectal tumor (Ma, 2019), TLG is often combined with other Chinese medicine for clearing heat toxicity for the treatment of CRC, gastric cancer, liver cancer, and other digestive system tumors (Gu, 2012; Qi and Qing, 2014). It was found in clinical studies that TLG decoction with mFOLFOX6 regimen in the treatment of advanced CRC can enhance the immune function of patients, reduce toxic side effects, and improve the therapeutic effect (Su, 2018). TLG compound combined with chemotherapy can effectively reduce the recurrence of CRC by postoperative treatment of colon cancer (Fang, 2017). Modern pharmacological studies have shown that ursolic acid in TLG could inhibit the proliferation of colon cancer cells (Shan Jianzhen Mechanism study of active, 2010) and its extracts could induce the apoptosis of CRC HT-29 cells (Chen and Shi, 2012). The formulation of TLG compound can effectively inhibit artificial lung metastasis of colon cancer in mice (Guo and Guo, 2014), then its anti-CRC effect may be related to the regulation of intestinal flora (Zheng, 2017).

According to the theory of traditional Chinese medicine, the deficiency of healthy qi in the body and the accumulation of pathogenic dampness are one of the fundamental causes of CRC, while clearing heat and promoting dieresis is the main treatment for this type of CRC. It is an effective compatibility prescription for the prevention and treatment of damp–heat CRC with the function including strengthening and consolidating body resistance, invigorating spleen for eliminating dampness, and clearing heat toxicity. On basis of the earlier stage, this article intends to further clarify the prevention and treatment effect of the Huangqin Tang with TLG on the type of damp–heat CRC through the mice model of CRC induced by AOM and DSS, and preliminarily explain its mechanism.

## 2 Materials and Agents

### 2.1 Experimental Animals

Ninety-one male C57BL/6 mice (6 weeks old, weight 20–22 g) were purchased from the Animal Experiment Research Center of Zhejiang Chinese Medical University (provided by the Shanghai Slack Laboratory Animal Co., Ltd.); animals were raised in the Experimental Animal Center of Zhejiang Chinese Medical University (certificate number: 20170005051764). The mice were housed in a controlled environment (21 ± 2 C, 12-h light/dark cycle) for at least 1 week to adapt to the environment before experiments. This experiment strictly complied with the relevant regulations of the Laboratory Animal Management Ethics Committee of Zhejiang Chinese Medical University and conformed to animal ethics and moral standards.

## 3 Materials

*Ziziphus jujuba* Mill*.* (Shangdong, 210301), *Scutellaria baicalensis* Georgi (Shanxi, 210301), *Glycyrrhiza uralensis* Fisch*.* (Xinjiang, 210301), *Cynanchum otophyllum* Schneid*.* (Anhui, 210301), and *Actinidia arguta* (Sieb. et Zucc) Planch. ex Miq. (Zhejiang, 210201) were purchased from Zhejiang University of Traditional Chinese Medicine Piece Co., Ltd.

1) Preparation of Huangqin Tang: Take the medicinal materials of each group of Huangqin Tang (18 g of *Scutellaria baicalensis* Georgi*.*, 12 g of *Cynanchum otophyllum* Schneid*.*, 12 g of *Glycyrrhiza uralensis* Fisch*.*, and 9 g of *Ziziphus jujuba* Mill*.*), add 10BV pure water, extract at 100°C for 60 min, separate the filtrate after filtration; add 8BV pure water to the residue, extract at 100°C for 45 min, separate the filtrate after filtration, combine the two filtrates, and concentrate under reduced pressure to 0.8 g (crude drug)/ml, that is, the Huangqin Tang (HQT).
2) Take Tengligen (30 g), extract and concentrate with the above method to prepare the solution with a concentration of 0.5 g (crude drug)/ml (TLG).
3) Take the medicinal materials of each group of Huangqin Tang (18 g of *Scutellaria baicalensis* Georgi*.*, 12 g of *Cynanchum otophyllum* Schneid*.*, 12 g of *Glycyrrhiza uralensis* Fisch*.*, and 9 g of *Ziziphus jujuba* Mill*.*) and *Actinidia arguta* (Sieb. et Zucc) Planch. ex Miq. (30 g), add 10BV pure water, extract at 100°C for 60 min, after filtration, separate the filtrate; add 8BV pure water to the residue, extract at 100 °C for 45 min, filter, divide the filtrate, combine the two filtrates, and concentrate under reduced pressure to 1.2 g (crude drug)/ml (HQT + TLG).

Celecoxib capsules (Celebrex, Pfizer Pharmaceuticals LLC): 0.2 g, 6 capsules/box, manufacturer: common feed was provided by the Laboratory Animal Center of Zhejiang Chinese Medical University.

## 4 Methods

### 4.1 Mice and Groups

A model of CRC with dampness–heat accumulation was established by using azoxymethane (AOM, A5486, Sigma)–dextran sodium sulfate (DSS, S5036, MP Biomedicals) (Li, 2017; Cao and Zhou, 2020). From the first week of modeling, AOM (12.5 mg/kg) with a concentration of 1 mg/ml was intraperitoneally injected and 2.5% DSS was given free drinking water for 1 week and then every 2 weeks. The modeling was completed after a total of three to four cycles.

Ninety-one mice were randomly divided into 6 groups, 8 in the vehicle group (distilled water via gavage), 23 in the model group (distilled water via gavage), 15 in the celecoxib group (20 mg/kg/d via gavage), 15 in the HQT group (8 g/kg via gavage), 15 in the TLG group (5 g/kg via gavage), and 15 in the HQT-flavored TLG group (12 g/kg via gavage), and the treatment was started at the same time as the modeling. All animal experiments were approved by the Experimental Animal Ethics Committee of Zhejiang Chinese Medicine University.

### 4.2 ELISA Assay and Immunofluorescence Assay

Detection of tumor necrosis factor-α (TNF-α, H052-1, Nanjing Jiancheng), interleukin-6 (IL-6, H007-1-2, Nanjing Jiancheng), IL-10 (H009-1, Nanjing Jiancheng), IL-17 cytokine levels (H014-1, Nanjing Jiancheng) was carried out according to the ELISA kit instructions.

### 4.3 Pathological Analysis

Colorectal tissue was fixed with formaldehyde for 48 h, then dehydrated with graded alcohol, cleared in xylene, embedded in paraffin, cut into 4-μm thick sections using a microtome (HM335E, Microm, Inc.), and dewaxed and hydrated with hematoxylin–eosin staining, followed by neutral gum mounting to observe the pathological changes of colon tissue. Sections were stained with Masson’s trichrome method to observe the proliferation of submucosal collagen in colorectal tissue.

### 4.4 Immunohistochemistry Assay

Colorectal tissue paraffin sections were incubated with Ki-67 antibody (Ab16667, Abcam) overnight at 4°C, followed by HRP-labeled secondary antibody, then streptavidin–alkaline phosphatase solution was added for reaction, DAB for color development, and hematoxylin counterstaining. Each image was analyzed using image processing software.

### 4.5 TUNEL Assay

Paraffin sections were routinely dewaxed and hydrated, 20 μg/ml proteinase K at room temperature was added for reaction, and 50 μl of labeling reaction mixture (composed of 5 μl of TdT enzyme + 45 μl of labeling safety buffer, prepared and cooled on ice before use) was smeared at 37°C and humidified. Finally, incubate for 60 min and stop the reaction with PBS.

### 4.6 Flow Cytometry Analysis

The mesentery of each mouse was isolated and three to four lymph nodes in the upper mesentery were removed; rinse with PBS and triturate with a 40-μm cell mesh and the black rubber tip of a 1 ml syringe to filter the lymph nodes. Then, centrifuge at 400 *g* for 7 min at 4°C and discard the supernatant. Resuspend the pellet in 2 ml of erythrocyte lysate and lyse at 400 *g* on ice. Centrifuge for 7 min at 4°C, resuspend cells in buffer, and centrifuge. The cells were resuspended in PBS, then aliquoted, and the corresponding antibodies were added. Antibody (1 µL) (CD3/CD4/CD8/CD25) was incubated in the dark for 15 min and 100–300 μl of stain buffer was added to resuspend. The cells were detected by flow cytometry (CytoflexS, Beckman Coulter).

### 4.7 Quantitative Real-Time PCR Analysis

Total RNA was extracted with RNA extraction kit (AHF 1991D, TaKaRa); SYBR Premix Ex TaqTMⅡ (AK9301, TaKaRa) was used for amplification reaction, samples were analyzed on ABI StepOnePlus PCR machine, Ct value and transcription level (2^−△△CT^) were calculated. The primers were designed and synthesized by Sangon Bioengineering (Shanghai) Co., Ltd. The sequences are shown in Supplementary Table S2.

### 4.8 Western Blot Assay

Total protein was extracted from colorectal tissue, protein concentration was determined by BCA protein quantitative kit, and the protein expression level of NF-κB p65 (8242S, CST) and STAT3 (12640S, CST) was detected by automatic Western blot quantitative analysis system (Wes, Protein Simple).

### 4.9 16s DNA Sequencing

The mice feces were collected under sterile conditions, and the total genomic DNA of the fecal flora was extracted for PCR amplification. The primers correspond to the region: 16S V3–V4 region, the upstream primer 338F: ACT​C-CTA​CGG​GAG​GCA​GCA​G, and the downstream primer 806R: GGACTACHVG-GGTWTCTAAT. Agarose gel (2%) electrophoresis, AxyPrep DNA Gel Extraction Kit (Axygen Biosciences, Axygen) for purification of PCR products, NEXTFLEX Rapid DNA-Seq Kit (Bioo Scientific) for library construction, and MiSeq Reagent Kit v3 Illumina for sequencing were also carried out.

### 4.10 Statistical Analysis

All statistical analyses were performed with SPSS 25.0. All data are expressed as the mean ± standard deviation (SD). The differences between the groups were determined by using a two-tailed Student’s *t*-test and one-way ANOVA. Statistical significance was set at *p* < 0.05.

## 5 Results

### 5.1 The Combination of Huangqin Tang and Radix *Actinidiae chinensis* (HQT + TLG) Possessed a Positive Effect on Anti-Colorectal Cancer Induced By AOM and DSS Solution

In our study, the mice in the vehicle group were in good mental state, with normal food intake, normal drinking water, normal bowel movements, moist fur, and flexible movements. As the experiment progressed, their body weight gradually increased. Except for the vehicle group, on the 10th day after the modeling, the mice in the model group developed mucus, and some mice had poor mental state and no vitality; on the 12th day after the modeling, the mice in each group had different degrees of mucus, pus, and blood in the stool; at the 9th week of modeling, the mice in each group appeared anal prolapse one after another. At the end of the experimental period, the mice in each group showed various degrees of lack of energy, thin body, dull hair color, poor vitality, lumps, loose stools, and other symptoms of damp–heat accumulation. Death: During the experiment, from the 15th day, different numbers of mice died in each model group; by the end of the experiment, there were eight mice in the model group and the mortality rate was 65.2%; each tested drug could be different as the mortality rate of mice was reduced to a certain extent, and the mortality rate of mice in the HQT + TLG group was the lowest at 20.0% (Supplementary Table S3).

**FIGURE 1:**
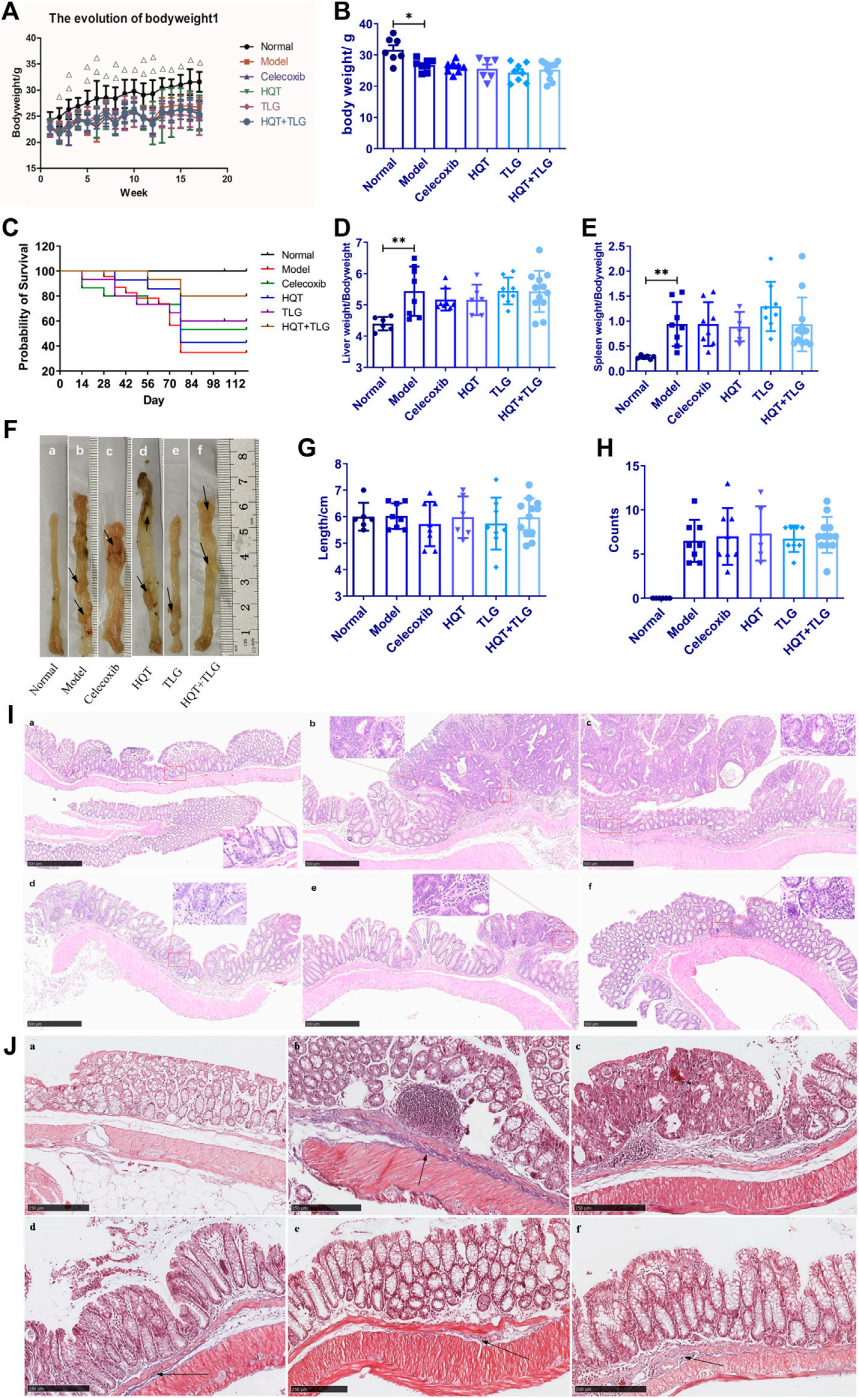
Effect of Huangqin decoction (HQT) combined with Radix *Actinidiae chinensis* (TLG) on colorectal cancer induced by AOM and DSS. **(A)** Body weight change curve of mice in each group. **(B)** Bodyweight at the end of experiment. **(C)** Survival curve of mice in each group. **(D)** Liver index. **(E)** Spleen index. **(F)** General view of colorectum. **(G)** Length of colon. **(H)** Number of colon tumors. **(I)** Representative photographs of HE staining. **(J)** Masson’s staining. **(A)** Normal control group (normal); **(B)** model control group (model); **(C)** celecoxib group (celecoxib); **(D)** Huangqin Tang (HQT) group; **(E)** Radix *Actinidiae chinensis* group (TLG); **(F)** HQT + TLG group. Compared with the normal control group, *p < 0.05, **p < 0.01.

After modeling, as the body weight of each model group consistently decreased, the vehicle group increased steadily (Figure 1A, *p* < 0.01). At the end of the experiment, the body weight of the mice in the model group was lower than the vehicle group (*p* < 0.05). Compared with the model group, there was no significant difference on the body weight between each treated group. Compared with the vehicle group, the liver and spleen index of the model group increased dramatically (*p* < 0.01). Compared with the model group, the liver and spleen index of the mice in the Huangqin Tang intervention group had no obvious downward trend (Figures 1D,E).

### 5.2 Huangqin Tang Ameliorates Colorectal Pathology in Mice

To further discuss the effect of Huangqin Tang on CRC, we observed the colonic pathological results in six groups of mice. In the model group, the tumor formation rate was 100%, it is detected that the tumors protruded into the intestinal cavity as a polypoid and primarily located in the distal colon. Compared with the model group, the number of tumors decreased in the mice of TLG and HQT + TLG groups (Figures 1F,H). The results of HE staining showed that the intestinal mucosal cells and crypts in the carrier group had regular structures. The model group was obviously disordered, with atypical cells, huge and hyperchromatic nuclei, pathological mitosis, and high-grade intraepithelial neoplasia. Local intramucosal carcinoma is formed, tumor cells are significantly different in cell size and morphology, and a large number of inflammatory cells are infiltrated in the interstitium. The intestinal mucosa of mice in HQT group, TLG group, and HQT + TLG group was relatively intact. However, some glandular villous adenomas, low-grade intraepithelial neoplasia, and granulation tissue hyperplasia were seen in the intestinal tissues of mice in the HQT group and TLG group, and nodular lymphocyte aggregation was seen in the surrounding tissues of the intestines.

The results of Masson’s staining revealed that the submucosal collagen hyperplasia in the intestinal tissue of the mice in the model group was obvious, the annular muscle layer and the longitudinal muscle layer were thickened, and the base inflammatory cell infiltration increased; the colon tissue of the HQT group mice was improved. The proliferation of submucosal collagen in the intestinal tissue decreased in the TLG group, while the thickening of annular muscle and longitudinal muscle layer in the HQT + TLG group was significantly improved (Figures 1I,J).

### 5.3 Effects of Huangqin Tang on the Proliferation and Apoptosis of Colorectal Cancer

**FIGURE 2:**
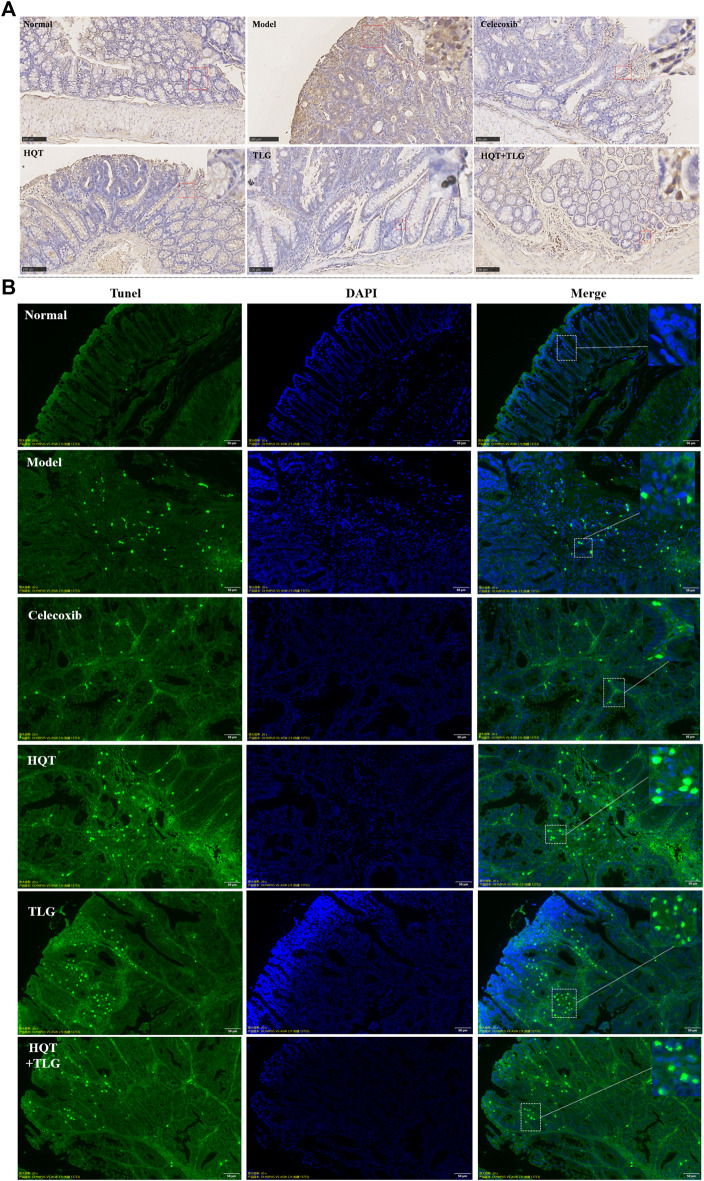
Effect of HQT + TLG on colorectal cancer cells’ proliferation and apoptosis. **(A)** Expression of Ki-67 in colorectal tumor tissues of mice in each group (DAB staining). **(B)** Apoptosis of tumor cells in colorectal tumor tissue of mice in each group.

Ki-67 is a sensitive cell proliferation-associated nuclear antigen. Abnormally expressed in various tumors and precancerous lesions, it is closely related to the occurrence, development, and metastatic potential of cancer. The results of Figure 2A show that the intestinal epithelial cells of the vector group did not have positive expression of Ki-67. There were more cells with high expression of Ki-67 in the edge of colorectal tumor in the model group. Compared with the model control group, there were fewer Ki-67-positive expressing cells in the colorectal tumor tissue and adjacent tissue of the HQT, TLG, and HQT + TLG mice. The Ki-67-positive cells in the HQT + TLG group were mainly distributed near the lamina propria. The results of TUNEL staining showed that a small number of tumor cells were apoptotic in the colorectal tumor tissue of the mice in the vehicle group. Compared with the model group, the number of apoptotic cells in the colorectal tumor tissue of mice in the HQT, TLG, and HQT + TLG groups increased, suggesting that each drug could induce tumor cell apoptosis (Figure 2B).

### 5.4 Huangqin Tang Reduces the Expression Level of Inflammatory Cytokines in Colorectal Tissue

**FIGURE 3:**
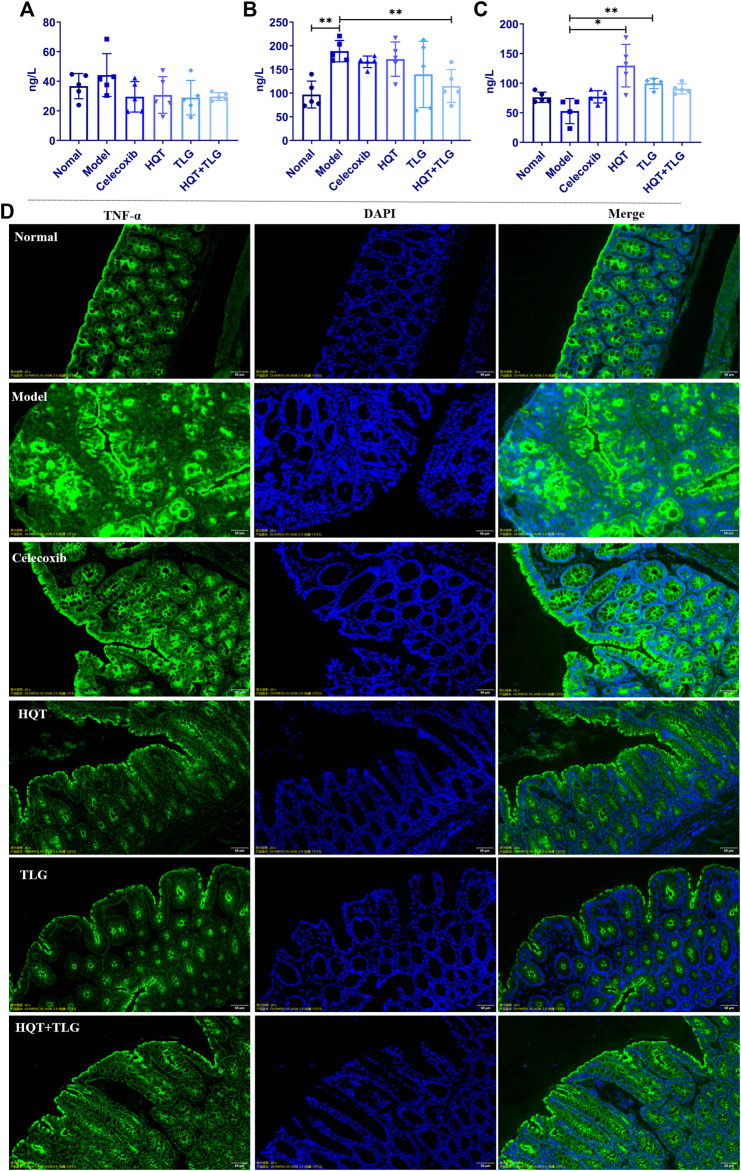
The levels of inflammatory factors and the expression of TNF-α in colon tissue of mice in each group. The levels of **(A)** IL-6, **(B)** TNF-α, and **(C)** IL-10 in the colorectum of the mice. **(D)** The expression of TNF-α in colon tissue of mice. Compared with the model control group, *p < 0.05, **p < 0.01.

The results of ELISA kit on colorectal tissue (Figures 3A–C) showed that compared with the vehicle group, the levels of IL-6 and TNF-α cytokines in the colorectum of the mice in the model group were significantly increased, the level of TNF-α was significantly different (*p* < 0.01), and the level of IL-10 decreased distinctly. Compared with the model group, the level of IL-6 and TNF-α in the colorectal tissue of the mice in each administration group were decreased to varying degrees, and the level of IL-10 was significantly increased. Among them, the level of TNF-α in colorectal tissue of mice in the HQT + TLG group was significantly decreased (*p* < 0.01), and the level of IL-10 in the HQT and the TLG groups was significantly increased (*p* < 0.01, *p* < 0.05).

### 5.5 Effects of Huangqin Tang on Colorectal Lymphoid Tissue and Spleen T Cell

**FIGURE 4:**
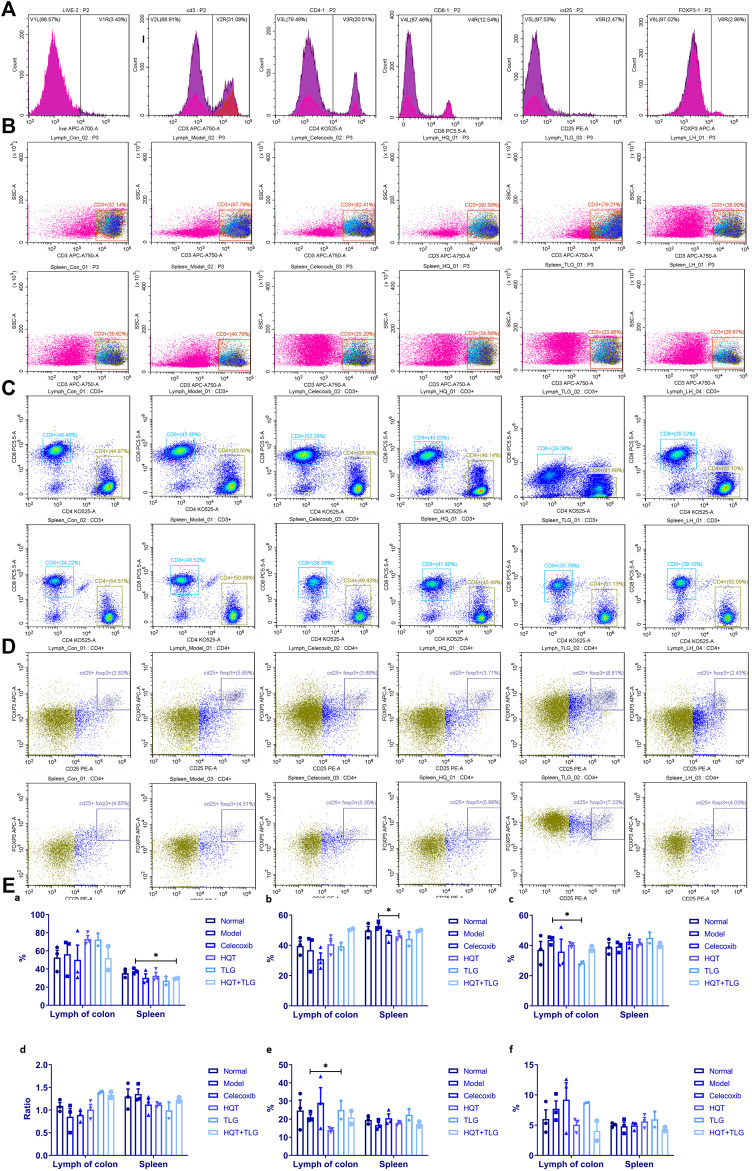
Results of T-cell analysis in colon tissue and spleen. **(A)** Single-tube staining. **(B)** Composition of T cells in intestinal lymph nodes. **(C)** Composition of T cells in spleen. **(D)** Composition of CD25^+^ FOXP3^+^ T cells in intestinal lymph nodes. **(E)** Statistics of T-cell classification. Compared with the model control group, *p < 0.05.

Figure 4 shows that compared with the vehicle group, there was no significant difference in the total number of CD3^+^ T cells in the large intestinal lymphoid tissue and spleen in the model control group, the ratio of CD3^+^/CD4^+^ T cells decreased, the ratio of CD3^+^/CD8^+^ T cells increased, and the ratio of CD4/CD8 declined. Compared with the model group, the total number of CD3^+^ T cells in the large intestinal lymphoid tissue of the mice in the HQT, TLG, and HQT + TLG groups increased. The ratio of CD3^+^CD4^+^ T cells and the ratio of CD4/CD8 also increased, while the ratio of CD3^+^CD8^+^ T cells decreased, and the effect was more significant in the TLG group (*p* < 0.05). The total number of CD3^+^ cells in the spleen of the HQT + TLG group was significantly less than that of the model control group (*p* < 0.05), and the proportion of CD3^+^CD4^+^ T cells in the spleen of the HQT mice was significantly less than that of the model control group (*p* < 0.05). Compared with the vehicle group, the proportion of CD3^+^CD4^+^CD25^+^ T lymphocytes in the large intestinal lymphoid tissue and spleen tissue in the model group decreased, and the proportion of CD3^+^CD4^+^CD25^+^Foxp3^+^ T lymphocytes in the large intestinal lymphoid tissue increased. Compared with the model group, the proportion of CD3^+^CD4^+^CD25^+^ cells in the large intestinal lymphoid tissue of the mice in the TLG group was significantly increased (*p* < 0.05). The proportion of these two types of cells was significantly decreased in the colorectal lymphoid tissue of HQT mice, while the proportion was increased in the spleen tissue.

### 5.6 Huangqin Tang Protects Against DSS + AOM-Induced Colorectal Cancer in Mice By Regulating IL-6/STAT3 Signaling Pathway

**FIGURE 5:**
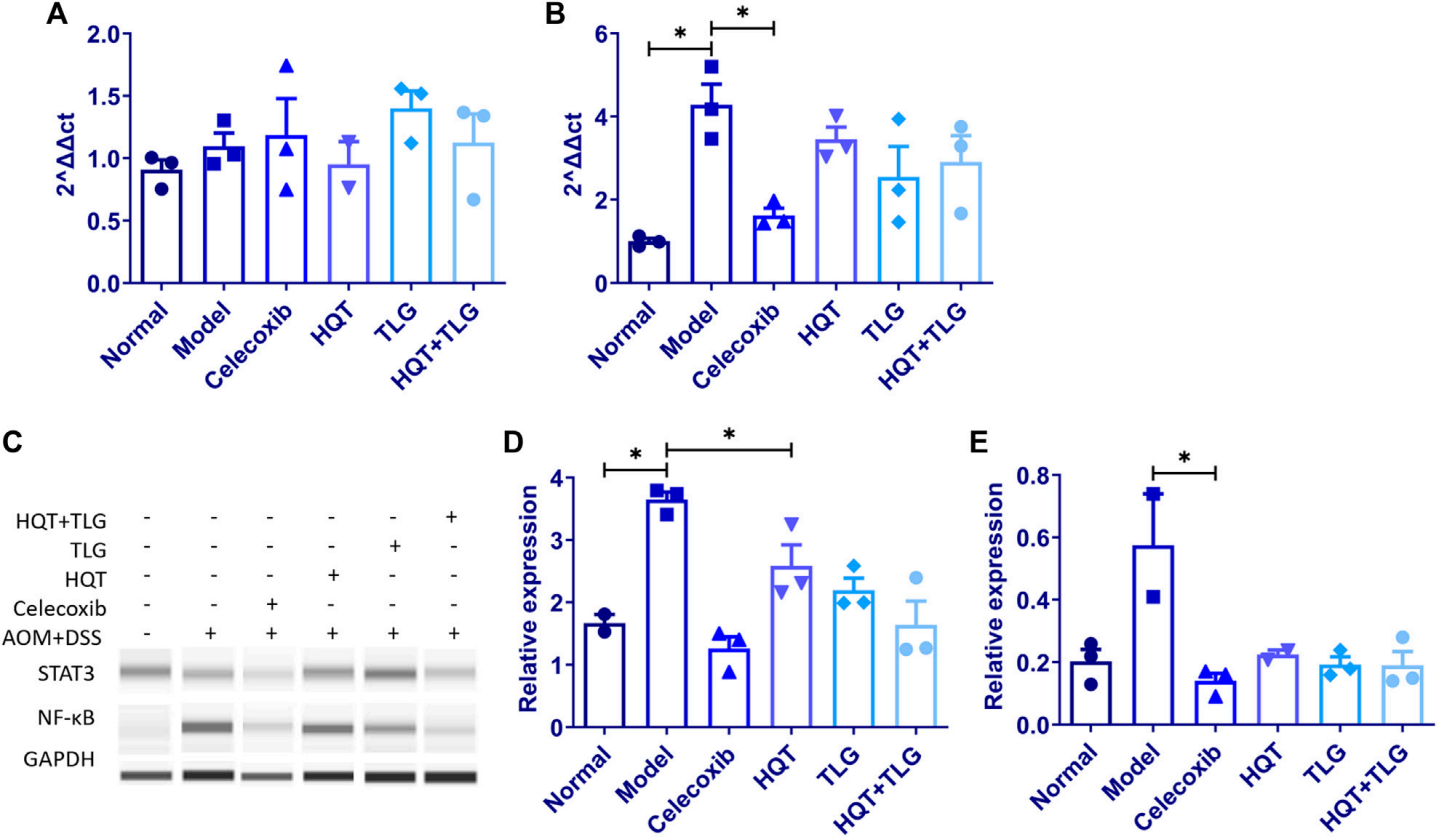
The mRNA expression of IL-6 and IL-17, and the protein expression of STAT3 and NF-κB in colon tissue of mice in each group. **(A,B)** Expression levels of IL-6 and IL-17 mRNA. **(C–E)** Expression levels of STAT3 and NF-κB protein. Compared with the model control group, *p < 0.05.

RT-PCR results showed (Figures 5A,B) that compared with the normal control group, the expression level of IL-6 mRNA in the colorectal tissue of the model control group showed an increasing trend and the expression level of IL-17 mRNA was significantly upregulated (*p* < 0.05). Compared with the model control group, the expression level of IL-6 mRNA in the colorectal tissue of the mice in the HQT group decreased to some extent and the expression level of IL-17 mRNA in the colorectal tissue of the mice in the HQT, TLG, and HQT + TLG groups all decreased to varying degrees; the role of TLG and HQT + TLG is more obvious.

The results of Western blot (Figure 5C) showed that compared with the vehicle group, the expression level of STAT3 and NF-κB in the colorectal tissue of mice in the model group was significantly upregulated and the difference in STAT3 was statistically significant (*p* < 0.05). The expression level of STAT3 and NF-κB in colorectal tissue of mice in the HQT, TLG, and HQT + TLG groups decreased in varying degrees (*p* < 0.05), and the effect of HQT + TLG group was more significant.

### 5.7 Effects of Huangqin Tang on the Expression of mRNA and Protein in the Colorectum of Model Mice

**FIGURE 6:**
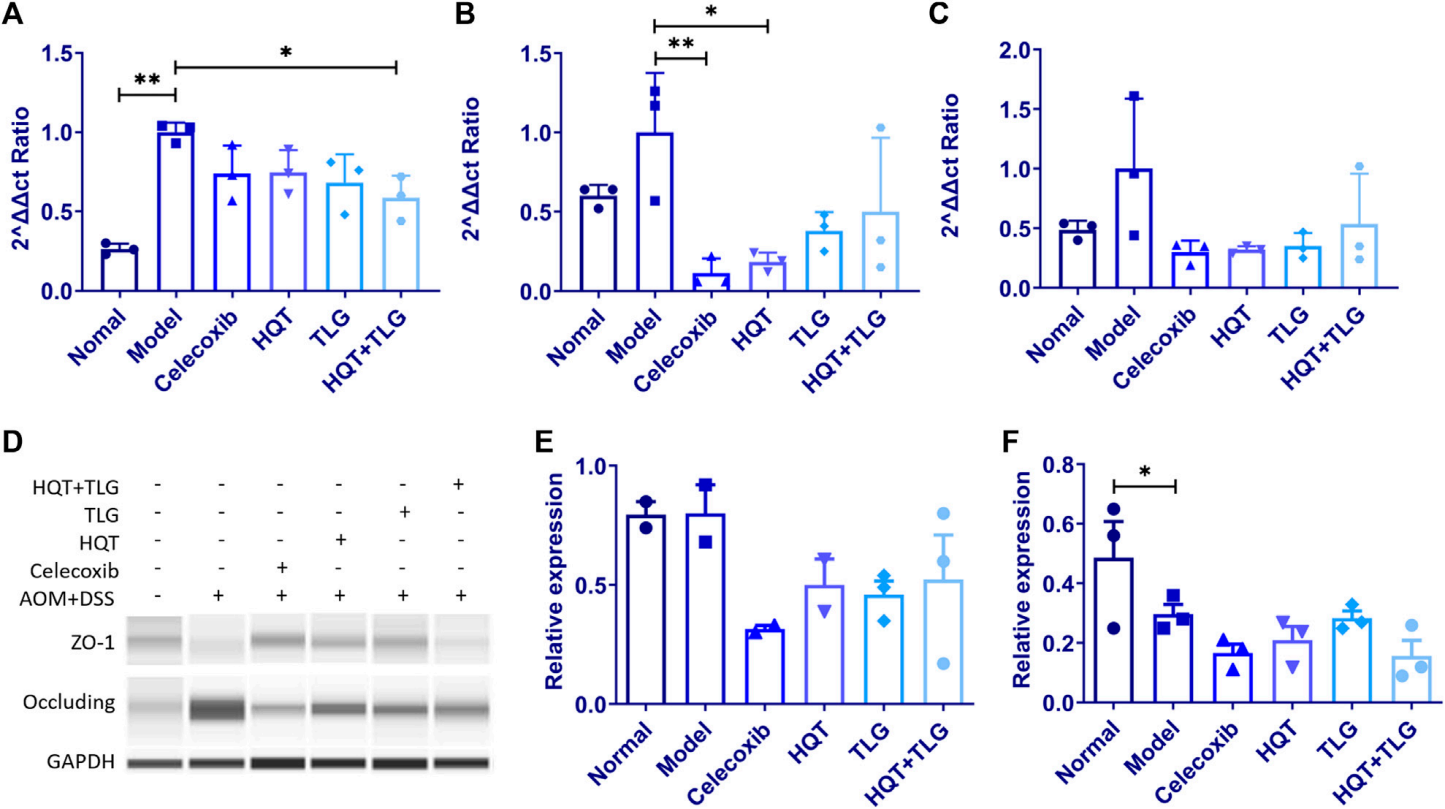
The expression levels of intestinal claudin-1, occludin, and ZO-1 mRNA and protein in mice in each group. **(A–C)** Expression levels of claudin-1, occludin, and ZO-1 mRNA. **(D–F)** Expression levels of occludin and ZO-1 protein. Compared with the model control group, *p < 0.05.

The results in Figures 6A,B show that compared with the vehicle group, the mRNA expressions of claudin-1 and occludin in the intestinal tight junction of mice in the model control group were significantly increased (*p* < 0.01, *p* < 0.05) and ZO-1 mRNA (Figure 6C) had an upward trend; compared with the model control group, the mRNA expression levels of claudin-1, occludin, and ZO-1 in the intestinal tissues of mice in the HQT, TLG, and HQT + TLG groups were decreased to varying degrees. The expression of occludin mRNA in Huangqin Tang group was significantly decreased (*p* < 0.05). Compared with the normal control group, the expression of occludin protein in the colorectal tissue of the mice in the model control group was upregulated and the expression level of ZO-1 protein was significantly decreased (*p* < 0.05). There was no significant difference in occludin protein expression and ZO-1 protein expression level, but the expression level in the HQT + TLG group was relatively low (Figures 6D–F).

### 5.8 Effects of Huangqin Tang on Intestinal Microflora

**FIGURE 7:**
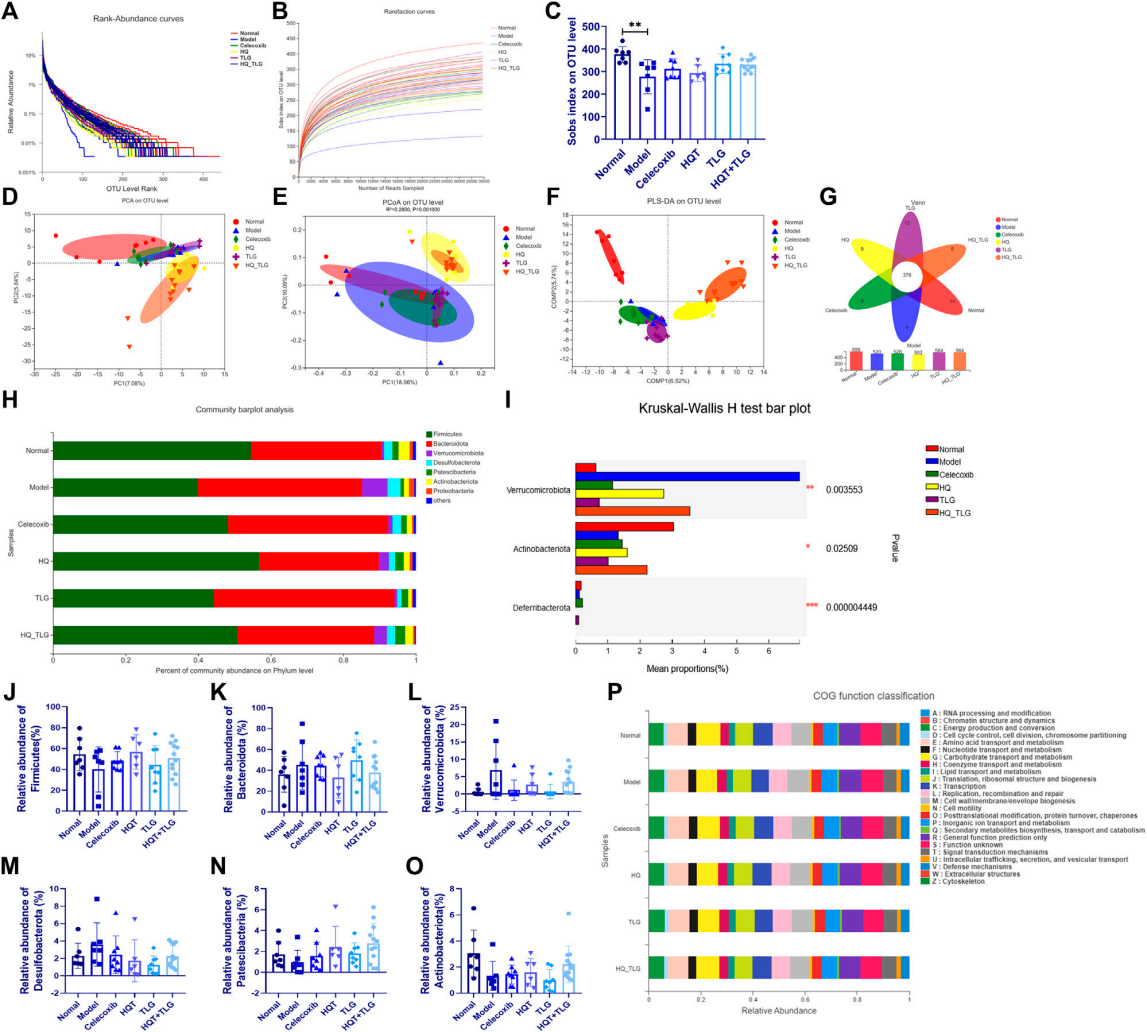
Intestinal flora analysis and gene function classification. **(A–C)** Annotation and assessment of flora species. **(D–O)** Difference analysis of flora species. **(P)** Gene function classification.

In this study, the more intuitive rank abundance curve is used to describe the OTU classification. As shown in Figure 7A, the vehicle group has the largest range on the horizontal axis, the model group flora curve is steeper, and the species diversity of its intestinal flora is the lowest, suggesting that the intervention of AOM and DSS solution can affect the intestinal TLG of mice bacterial structure. As shown in Figure 7B, the abscissa is the amount of randomly selected sequencing data and the ordinate is the observed number of species at the selected taxonomic level. The curves of each group in the figure tend to be flat, indicating that the amount of sequencing data is large enough, the quantity is reasonable, and the sample size is sufficient to reflect the vast majority of microbial diversity information in the experimental samples. The Sobs index curve analysis found that model was at the bottom, indicating that at the OUT classification level, this group of samples contained the least number of species. In the statistical analysis of Sobs index, the higher the Sobs index, the higher the flora richness. As shown in Figure 7C, compared with the normal group, the diversity of the intestinal flora of the mice in the model group was significantly reduced (*p* < 0.01); compared with the model group, the intestinal flora of the mice in each administration group was diverse. The TLG and HQT + TLG groups increased relatively significantly.

As shown in Figures 7A–C, the results of PCA, PCoA, and PLS-DA showed that the intestinal flora of mice in the model group was significantly different from that of the vehicle group. In comparison, the intestinal flora of mice in HQT and HQT + TLG groups changed significantly, but there was still a big difference compared with the vehicle group. Venn diagrams can be used to count the number of common and unique species (such as OTUs) in multiple groups or samples and can intuitively represent the similarity and overlap of the number of species OTUs in the environmental samples. As can be seen from Figure 7G, the total number of OTUs in the intestinal flora of mice in each group is 376, the number of OTUs in the normal control group is at most 595, and the number of OTUs in the HQT group is at least 502. The OTU numbers of the intestinal flora of mice in the TLG and HQT + TLG groups were close to those in the normal control group.

The results of species composition analysis at the phylum level showed (Figures 7J–O) that there were six species with a relative abundance of more than 0.1% in the intestinal flora of mice in each group, namely Firmicutes, Bacteroidetes, Verrucomicrobium, Desulfobacterota, Patescibacteria, and Actinobacteriota. Firmicutes and Bacteroidetes in the intestinal flora of mice in each group were all for the dominant bacterial phylum. Compared with the vehicle group, the abundance of bacteria such as Firmicutes and Actinobacteriota in the model group decreased significantly, while the bacterial groups such as Bacteroidetes, Verrucomicrobium, and Desulfobacterota were dramatically increased; compared with the model group, the abundance of Verrucomicrobium and Desulfobacterota in the celecoxib group was dramatically decreased.; the abundance of Firmicutes, Patescibacteria, and Actinobacteriota in the HQT + TLG group increased; and the abundance of Bacteroidota, Verrucomicrobiota, and Desulfobacterota increased significantly; the structural composition of the intestinal flora tended to be the vehicle group.

COG (clusters of orthologous groups of proteins) functional annotation refers to the functional annotation of unknown sequences through known proteins. The results in Figure7P show that, compared with the vehicle group, the model group has significant changes in carbohydrate transport and metabolism, transcription, cell membrane biogenesis, inorganic ion transport, and metabolism.

## 6 Analysis and Discussion

CRC is one of the most common gastrointestinal malignancies and dampness–heat syndrome is its primary TCM phenotype. According to TCM theory, Huangqin Tang (HQT) and Tengligen (TLG) can improve dampness–heat accumulation, and recent studies have shown that both medications have anti-tumor effects. HQT originates from the “Treatise on Cold Pathogenic Diseases” of Zhang Zhongjing, a famous traditional Chinese medicine physician in Chinese history, and recorded the effect of clearing heat and dampness, strengthening the spleen Qi. In Asia, TCM physicians use HQT to treat intestinal diseases, including dysentery, diarrhea, and CRC with dampness–heat syndrome. TLG is also an effective medicine against intestinal tumors and is widely used in gastrointestinal tumor clinical treatment. To further clarify the effectiveness and potential mechanism of the combination of the above drugs, we established the CRC mouse model by AOM + DSS. HQT and TLG were used as medical treatment for 6 weeks. The results of the experiments showed that the intervention of HQT, TLG, and the combination could effectively inhibit the progression of CRC and alleviate dampness–heat symptoms in mice.

Inflammatory bowel disease (IBD), such as ulcerative colitis (UC) and Crohn’s disease (CD), has been suggested to be associated with the occurrence of CRC (Zhang, 2020). CRC originates from inflamed mucosa and develop in the cascade of inflammation and cancer progression (Terzić et al., 2010; Zhang, 2020). In this pathological transformation, chronic inflammation plays a crucial role. Studies have shown that chronic inflammation factors such as TNF-α and interleukins contributes to tumorigenesis by driving oncogenes, modifying epigenetics, promoting cell proliferation, and supporting angiogenesis (Terzić et al., 2010; Li, 2017). For example, IL-6 inhibits apoptosis in an inflammatory environment (Hodge et al., 2005; Terzić et al., 2010) and promotes intestinal epithelial cell proliferation and tumor cell proliferation (Grivennikov et al., 2009). IL-17 is involved in the intestinal inflammatory immune responses and the development of various tumor processes such as CRC by increasing IL-6 and MCP-1 secretion by colonic fibroblasts (Hurtado et al., 2018; XU, 2019). Our results also showed that both HQT and TLG can reduce IL-6 and TNF-α levels and NF-κB protein expression in colorectal tissues and improve chronic inflammatory symptoms in the intestine. This implies that the mechanism of HQT and TLG is related to improving the chronic inflammatory microenvironment.

Furthermore, we tried to find the underlying signaling pathways related to the regulation of inflammation by the abovementioned drugs. Among them, STAT3 is a key cellular transcription factor and is often used as one of the biomarkers for tumor therapy and prognosis. STAT3 mediates the overexpression of inflammatory factors and inhibits tumor-specific immune responses, thereby inducing tumor growth. Therefore, the activation of endogenous STAT3 is one of the mechanisms of malignant tumor cell proliferation. Abnormal expression of NF-κB is also closely related to tumor cell migration, spread, and metastasis. Sustained activation of NF-κB will lead to unregulated proliferation of cells, thereby promoting tumor growth. STAT3 is a crucial signaling pathway mediated by IL-6. Studies have revealed that IL-6 promotes the activation of STAT3, and the expression of STAT3 in CRC showed a positive correlation with IL-6 (Zhang, 2017). The inhibition of IL-6 can block the continuous activation of STAT3 in cancer cells and can effectively prevent the development of CRC (Wang et al., 2013). IL-10 is a common anti-inflammatory cytokine; its expression level is negatively correlated with inflammatory response, and it plays a significant role in maintaining the normal immune regulation of intestinal mucosa. NF-κB can regulate the expression of various proinflammatory factors and is a key regulator in the process of inflammatory response, mainly involved in the regulation of immunity, inflammation, and cytokine proliferation and apoptosis. After being activated, NF-κB is involved in the activation of inflammatory cells, thereby regulating the expression of IL-10. Our findings also suggest that HQT, TLG, and the combination could reduce IL-6 and STAT3 protein expression. This suggests that the two drugs can regulate chronic inflammation by inhibiting IL-6/STAT3 signaling pathway, thus blocking CRC progression.

In addition to chronic inflammation, the immune microenvironment in which the tumor resides is likewise thought to be a vital regulator of tumor progression. Changes in immune cells, such as T lymphocytes and NK cells, can be used to monitor the immune status of CRC patients. It was found that the number of CD3^+^ T cells, CD3^+^CD4^+^ T cells, and CD4^+^/CD8^+^ ratio were significantly lower and the level of CD8^+^ cells was significantly higher in peripheral blood T cells of CRC patients compared to healthy population (Wang, 2020b). Not only that, alterations in peripheral blood T-lymphocyte subsets and NK cell numbers were positively correlated with the clinicopathological stage of CRC (SUN, 2021). It has been suggested that the level of infiltration of CD8^+^ T cells in the central region of tumor tissue can be used as a potential prognostic marker for CRC (Qi, 2021). The results of this study demonstrated that HQT and TLG increased the total number of CD3^+^ T cells, CD3^+^CD4^+^ T cell, and CD4^+^/CD8^+^ ratio in colonic lymphoid tissues and decreased the CD3^+^CD8^+^ T-cell ratio in CRC mice. TLG intervention alone also significantly increased the CD3^+^CD4^+^CD25^+^ T-lymphocyte ratio in spleen tissues of model mice, suggesting that the above herbal medicines can exert anti-tumor effects by regulating tumor immunity.

Activation of STAT3, in which IL-6 is involved, leads to a chronic inflammatory state and accelerates the progression of CRC. Studies have shown that the altered intestinal permeability caused by CRC is one of the leading causes of the release of inflammatory factors such as IL-6. For example, high expression of claudin-1 in CRC tissues has a proinflammatory effect on the colon and is positively correlated with TNF-α levels. This alteration induces epithelial–mesenchymal transformation and migration of tumor cells (Bhat et al., 2016). On this basis, we examined the expression of claudin-1, occludin, and ZO-1 before and after drug intervention to characterize the changes in intestinal permeability. In normal epithelium, claudin-1 is involved in maintaining epithelial cell polarity, regulating intestinal mucosal barrier permeability, and participating in the protective function of the body’s mucosal barrier. The expression of intestinal mucosal claudin-1 increases gradually with the progression of typical mucosa-polyp-adenoma-CRC, so claudin-1 is also one of the markers suggestive of early CRC (lin, 2007). Occludin was the first identified tight junction protein localized at tight junctions in intestine. Occludin regulates the diffusion of small hydrophilic molecules in the intercellular pathway and modulates the transepithelial migration of neutrophils (Peng, 2010). ZO-1 is a typical scaffolding protein that interacts with ZONAB to regulate gene expression and cell proliferation and morphological changes. Meanwhile, ZO-1 is able to interact with a variety of tight junction proteins such as occludin to link transmembrane proteins and cytoskeleton and intercellular tight junction assembly and function, thus regulating intra- and extracellular signal transduction pathways. Occludin and ZO-1 were lowly expressed in CRC patients (Cheng, 2017) and closely associated with postoperative cachexia in CRC (Wang, 2019). The results of this study showed that claudin-1 mRNA expression was significantly elevated and ZO-1 protein expression level was significantly decreased in the colon tissue of CRC model mice. HQT and TLG tend to reduce intestinal epithelial claudin-1 mRNA expression in model mice, but there was no significant difference in occludin and ZO-1 protein expression between the groups. These suggest that the reduction in chronic inflammation mediated by HQT and TLG is less relevant to the repair of the intestinal barrier.

In addition to the barrier of the intestinal mucosa, the disturbance of the intestinal flora and the release of intestinal metabolites are also important causes of the altered homeostasis of intestinal inflammation. The intestinal flora plays a vital role in maintaining intestinal physiological functions, participating in energy metabolism and regulating the immune function of the body. The restoration of the flora ecology can effectively inhibit the progression of CRC (Zhang et al., 2019). *Lactobacillus acidophilus*, *Bifidobacterium bifidum*, *Lactobacillus rhamnosus*, and *Streptococcus thermophilus* belonging to the phylum Firmicutes and the phylum Bacteroidetes are common probiotics in the intestinal flora, and they play important roles in regulating host metabolism, nonspecific immunity, and specific immune response. *Helicobacter pylori*, *Salix fragilis*, *Enterococcus faecalis*, *Clostridium septicum*, *Clostridium perfringens*, and pathogenic *Escherichia coli* have been identified as pathogenic bacteria that promote the development of CRC (Sun et al., 2019). The results of large-cohort clinical study also showed significant alterations in the gut microbiome of patients during the progression from multiple polypoid adenomas to intramucosal carcinomas and CRC development (Yachida et al., 2019). The results of our study showed that Firmicutes and Bacteroidetes were the dominant phylum in the intestinal flora of all groups of mice, but the model mice had decreased abundance of intestinal flora, especially in Firmicutes and Actinobacteriota. In the meantime, Bacteroidetes, Verrucomicrobium, and Desulfobacterota were upregulated. HQT intervention could increase the abundance of Firmicutes and Patescibacteria flora and improve the structure of intestinal flora. And further functional analysis showed that HQT combination with TLG could interfere with the biosynthesis, transport, and catabolism of metabolites in CRC mice through flora. This is closely related to the presence of chronic inflammation, as aberrant metabolism such as abnormal lipid metabolism can mediate the chronic inflammatory response of the body and promote the formation of tumor inflammatory microenvironment by enhancing oxidative stress (Vílchez et al., 2014). The COG database can analyze the proportion of proteins in different functional classes, reflecting the metabolic or physiological bias in the corresponding period and environment. Numerous studies have proven that the host and gut microbiome shape each other, and the imbalance of the gut microenvironment is deteriorated during the morbid state, which damages its beneficial effects immensely. Our COG function prediction showed that the improvement of CRC model by HQT + TLG may be completed through the metabolic pathway, with a certain metabolic bias. Alterations in carbohydrate metabolism in the gut microbiota may influence the expression of carbohydrate antigens in cancer. It also affects the production of intestinal glucose, which in turn affects the glucose metabolism of tumor cells and promotes tumor growth.

In conclusion, cumulative evidence supports that HQT with TLG exert anti-tumor effects by improving intestinal homeostasis and regulating the chronic inflammatory state in CRC mice through modulating the flora structure. Our study provides a new alternative for the treatment of CRC, especially for those CRC patients with dampness–heat syndrome. We also initially revealed the underlying mechanism of its action. However, we have to admit that there are still many shortcomings in this study. How the alteration of flora structure regulates the formation and spread of inflammation explicitly is not revealed by the present results yet. Due to the complex composition and numerous targets of herbs, we need to continue to explore the active ingredients in depth in the subsequent experimental studies and carry out the next phase of the task with numerous approaches.

## Data Availability

The datasets presented in this study can be found in online repositories. The names of the repository/repositories and accession number(s) can be found at https://www.ncbi.nlm.nih.gov; PRJCA009427.
