# Supplementary figures and images for: Synergistic Effect of Huangqin Decoction Combined Treatment With Radix Actinidiae chinensis on DSS and AOM-Induced Colorectal Cancer
Source: Front Pharmacol. 2022 Jul 6;13:933070. doi: 10.3389/fphar.2022.933070 (PMC9301036; doi:10.3389/fphar.2022.933070)

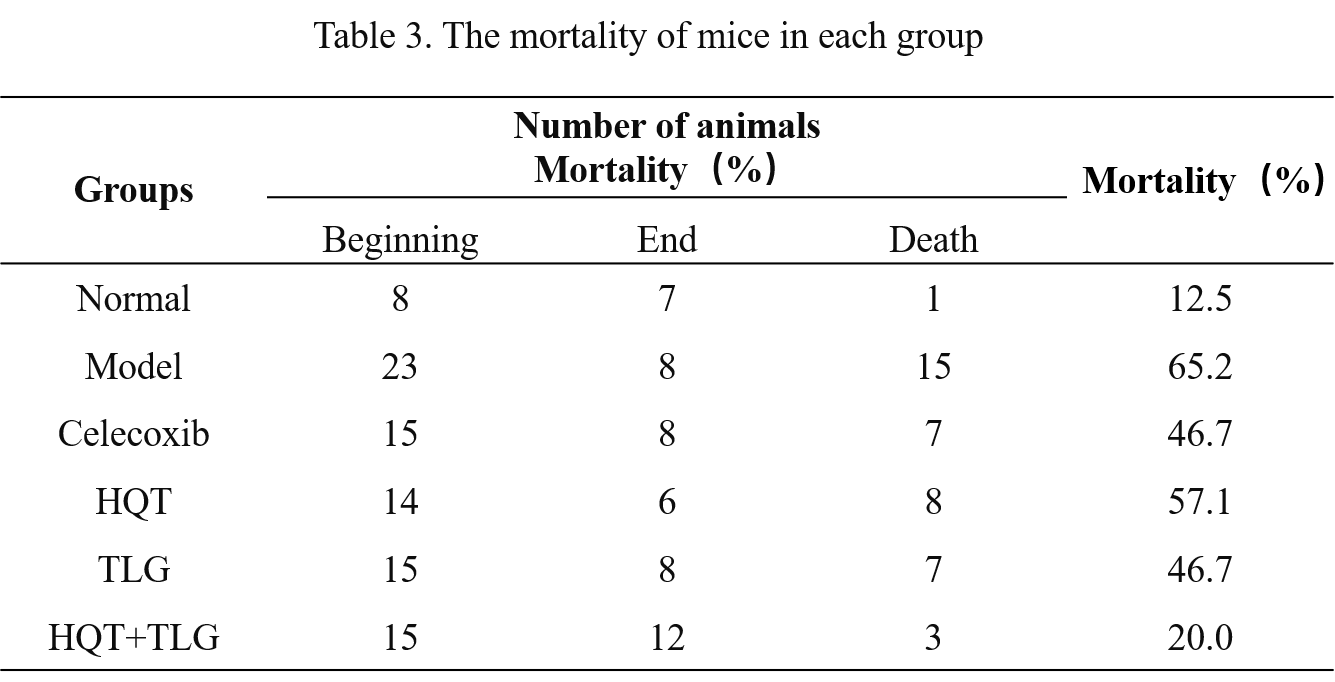

Supplement: Supplementary file 1 [file Image3.TIF]

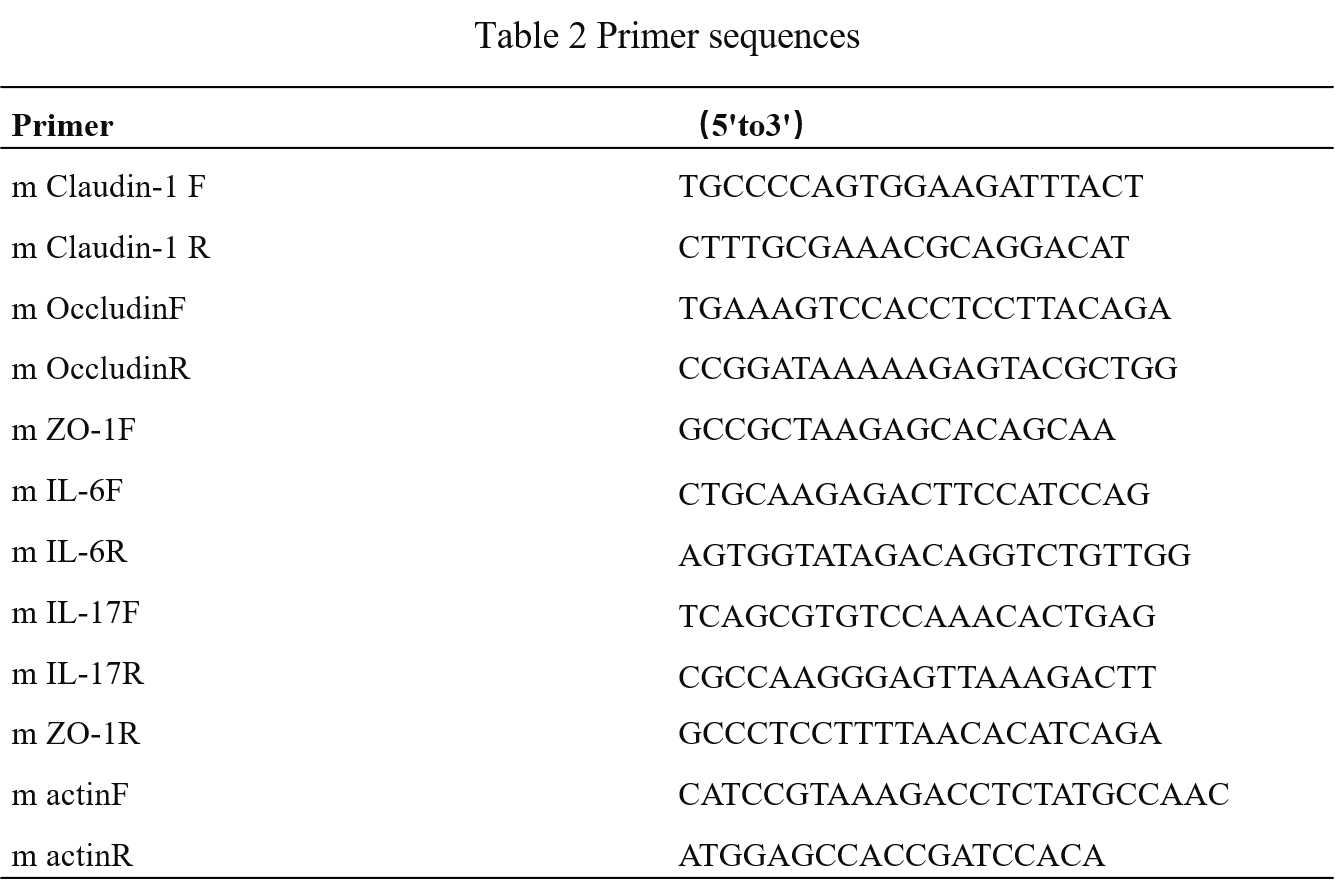

Supplement: Supplementary file 2 [file Image2.TIF]

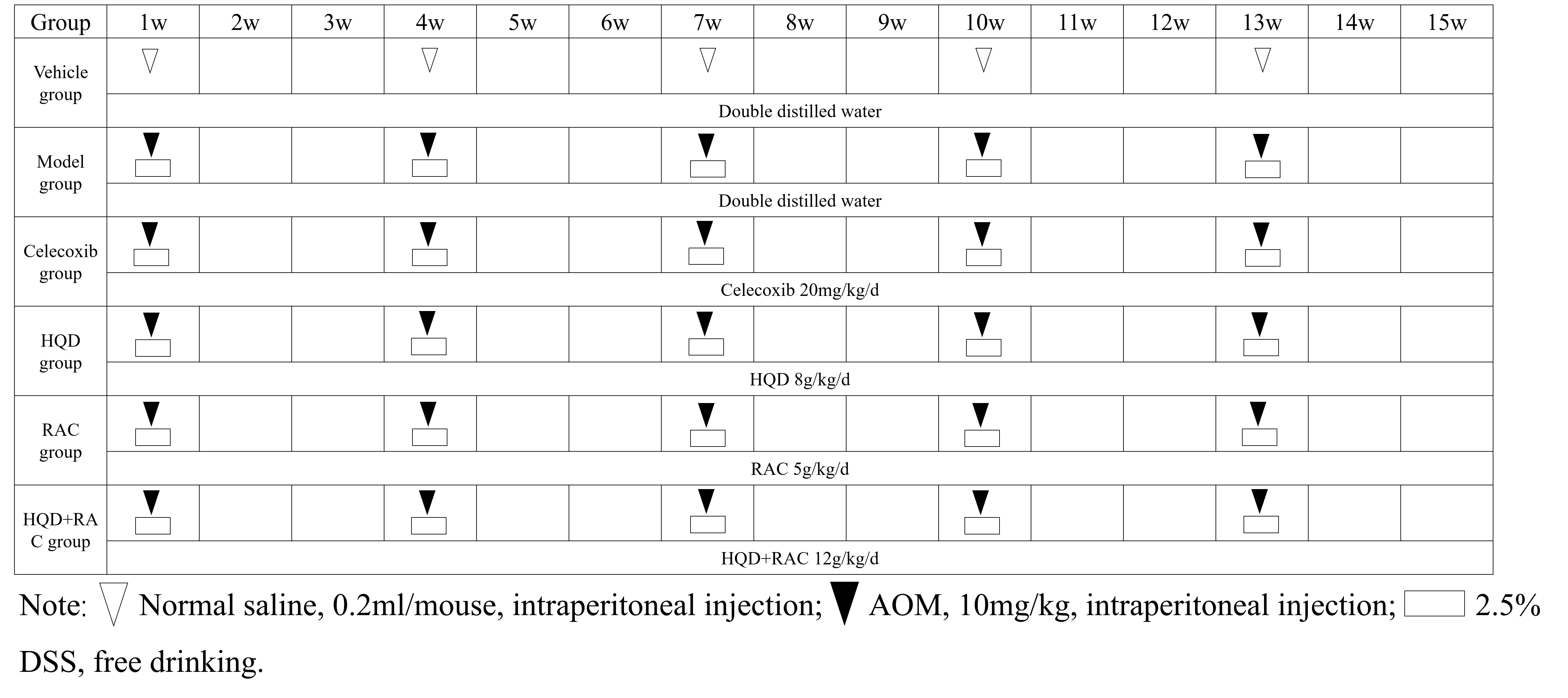

Supplement: Supplementary file 3 [file Image1.TIF]
